# Life-course determinants of bone mass in young adults from a transitional rural community in India: the Andhra Pradesh Children and Parents Study (APCAPS)^1^,^2^,^3^

**DOI:** 10.3945/ajcn.113.068791

**Published:** 2014-04-02

**Authors:** Mika Matsuzaki, Hannah Kuper, Bharati Kulkarni, KV Radhakrishna, Heli Viljakainen, Amy E Taylor, Ruth Sullivan, Liza Bowen, Jon H Tobias, George B Ploubidis, Jonathan C Wells, Dorairaj Prabhakaran, George Davey Smith, Shah Ebrahim, Yoav Ben-Shlomo, Sanjay Kinra

**Affiliations:** 1 From the Departments of Non-communicable Disease Epidemiology (MM, RS, SE, and SK), the Department of Medical Statistics (GBP), and Clinical Research (HK), London School of Hygiene and Tropical Medicine, London, United Kingdom; the Institute of Health and Biomedical Innovation, Queensland University of Technology, Brisbane, Australia (BK); the National Institute of Nutrition, Hyderabad, India (BK and KVR); the Musculoskeletal Research Unit, School of Clinical Sciences (HV and JHT) and the School of Social and Community Medicine (AET, YB-S, and GDS), University of Bristol, Bristol, United Kingdom; St George's University, London, United Kingdom (LB); the Childhood Nutrition Research Centre, UCL Institute of Child Health, London, United Kingdom (JCW); the Centre for Chronic Disease Control, New Delhi, India (DP); and the South Asia Network for Chronic Disease, Public Health Foundation of India, New Delhi, India (SE).

## Abstract

**Background:** Undernutrition and physical inactivity are both associated with lower bone mass.

**Objective:** This study aimed to investigate the combined effects of early-life undernutrition and urbanized lifestyles in later life on bone mass accrual in young adults from a rural community in India that is undergoing rapid socioeconomic development.

**Design:** This was a prospective cohort study of participants of the Hyderabad Nutrition Trial (1987–1990), which offered balanced protein-calorie supplementation to pregnant women and preschool children younger than 6 y in the intervention villages. The 2009–2010 follow-up study collected data on current anthropometric measures, bone mineral density (BMD) measured by dual-energy X-ray absorptiometry, blood samples, diet, physical activity, and living standards of the trial participants (*n* = 1446, aged 18–23 y).

**Results:** Participants were generally lean and had low BMD [mean hip BMD: 0.83 (women), 0.95 (men) g/cm^2^; lumbar spine: 0.86 (women), 0.93 (men) g/cm^2^]. In models adjusted for current risk factors, no strong evidence of a positive association was found between BMD and early-life supplementation. On the other hand, current lean mass and weight-bearing physical activity were positively associated with BMD. No strong evidence of an association was found between BMD and current serum 25-hydroxyvitamin D or dietary intake of calcium, protein, or calories.

**Conclusions:** Current lean mass and weight-bearing physical activity were more important determinants of bone mass than was early-life undernutrition in this population. In transitional rural communities from low-income countries, promotion of physical activity may help to mitigate any potential adverse effects of early nutritional disadvantage.

## INTRODUCTION

Urbanization has been linked to the rise of many noncommunicable diseases (NCDs)^4^, including osteoporosis (1). A conservative estimate has suggested that ∼25 million Indian adults will have osteoporosis by 2015 (2). In rural India, rapid socioeconomic development is resulting in drastic changes in diet and activity patterns, which are key determinants of bone mass in young adults. Whereas the exact mechanisms underlying the association between urbanization and bone health are unclear, several potential risk factors have been identified, including a lower physical activity level, serum 25-hydroxyvitamin D [25(OH)D] concentration, and fruit and vegetable intake (3, 4).

The possibility of a developmental origin of osteoporosis has been suggested (5). A meta-analysis of observational studies found a weak positive association between adult bone mass and birth weight—a proxy measure for early-life nutrition (6). However, birth weight may be influenced by factors other than maternal nutrition, and direct evidence from nutritional trials is lacking (7).

There are many potential risk factors for bone mass accrual in adults. Previous studies have generally shown a positive association between bone mass accrual and weight-bearing physical activity (wbPA), whereas evidence on the effects of nutrition on bone mass is inconsistent (8–12). BMI, and also its main components of fat mass and lean mass, have been independently associated with bone mass accrual (13). Low serum 25(OH)D concentration and dietary intake of calcium, protein, and calories may contribute to poor bone health and osteoporosis (2, 14–18). How these adult risk factors play a role in bone mass accrual in young adults who have been experiencing significant nutritional and lifestyle transitions since their childhood is not well understood.

The Andhra Pradesh Children and Parents Study was established to understand societal and individual risk factors for NCDs in a transitional rural community. The study population experienced early-life undernutrition; two decades later, other lifestyle risk factors associated with rapid economic development are emerging (19, 20). This transition provided an opportunity to examine bone development in young adults whose bone mass may be influenced by both previous undernutrition and current urbanized lifestyles (21). In this manuscript, we present the analyses of life-course determinants of bone mass of young adults in this transitional rural community.

## SUBJECTS AND METHODS

### Study design

The analyses in this study used data from the second follow-up of the Andhra Pradesh Children and Parents Study, established through long-term follow up of the Hyderabad Nutrition Trial. The Hyderabad Nutrition Trial evaluated the Integrated Child Development Services scheme—a national community outreach program providing food supplementation along with health, hygiene, and nutrition education, immunization, anemia control, and basic health care to pregnant and lactating women and children younger than 6 y (22, 23).

The study received approvals from the ethics committees of the National Institute of Nutrition (Hyderabad, India), the Indian Council of Medical Research, and London School of Hygiene and Tropical Medicine (London, United Kingdom). Approval was also sought from the village heads and their committees in each of the 29 villages. The participants provided written informed consent, or a witnessed thumbprint if illiterate, before their inclusion in the study.

#### Initial trial (1987–1990) and first follow-up study (2003–2005)

**FIGURE 1.**
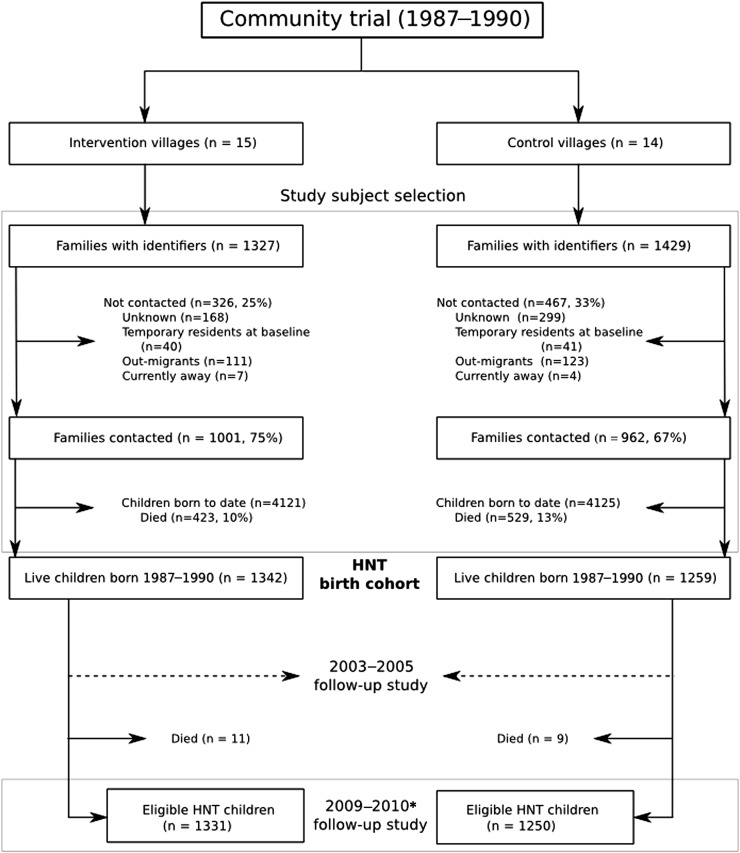
Flowchart of participant recruitment at follow-up in the Andhra Pradesh Children and Parents Study. *Includes people who migrated out of villages: intervention (*n* = 326), control (*n* = 366). HNT, Hyderabad Nutrition Trial.

A detailed description of the initial trial and the first follow-up study of the Hyderabad Nutrition Trial was previously published (24). Briefly, a controlled “stepped wedge design” trial was conducted in 1987–1990, using the opportunity afforded by the incremental expansion of Integrated Child Development Services program. A total of 29 villages in 2 adjacent administrative areas near Hyderabad City in India were selected: one with the Integrated Child Development Services program already in place (15 intervention villages) and the other awaiting implementation (14 control villages) (**Figure 1**). In the intervention villages, a nutritional supplement made of corn-soya blend and soybean oil was available daily to all pregnant and lactating women and children younger than 6 y. The meal (*upma*) contained, on average, 2.09 MJ and 20–25 g protein for pregnant and lactating women and ∼1.25 MJ and 8–10 g protein for children younger than 6 y. Supplementation was associated with a small but statistically robust (61 g; 95% CI: 18, 104 g; *P* = 0.007) increase in the birth weight of the offspring (24). The first follow-up study in 2003–2005 examined 1165 adolescents aged 13–18 y who were still resident in these villages. The adolescents in the intervention villages were 14 mm (95% CI: 4, 23 mm; *P* = 0.007) taller and had more favorable measures of insulin resistance and arterial stiffness, as evidenced by a 20% (95% CI: 3%, 39%; *P* = 0.02) lower HOMA score and 3.3% (95% CI: 1%, 5.7%; *P* = 0.008) lower augmentation index.

#### Second follow-up study (2009–2010)

The second follow-up study examined markers for chronic diseases affecting cardiovascular, musculoskeletal, and mental health in the cohort of young adults who took part in the initial trial. All consenting participants underwent clinical examinations at the National Institute of Nutrition, Hyderabad.

### Measurements

#### Questionnaire data

A semistructured questionnaire was administered to all participants by a trained interviewer. A subset of questions (14/29) from the Standard of Living Index (SLI), a summary measure of household level asset-based scale devised for Indian surveys, was used to estimate socioeconomic position as joint family structures are common in rural India (22). We collected information on the quality of house, toilet facilities, source of lighting and drinking water, and ownership of clock, radio, television, bicycle, motorcycle, car, refrigerator, telephone, and agricultural land. These items were weighted to give a maximum score of 34 by using weights developed by the International Institute of Population Science in India (22). Education was classified in 4 levels: no formal education, primary (1–4 y), secondary (5–12 y), and beyond secondary.

The food and physical activity questionnaires were developed and evaluated previously in this setting, and their performance was found to be satisfactory (25, 26). Dietary intake over the past year was estimated with a semiquantitative food-frequency questionnaire that collected information on the frequency of intake (daily, weekly, monthly, or yearly/never) of 98 commonly consumed food items. The nutrient content of a single portion of each food item on the list was estimated based on the Indian food-composition tables (27). Physical activity in the previous week was assessed across the following domains: work, travel, leisure (sports, games, exercise), household, and sedentary. For each activity, the average amount of time spent on the activity and the frequency of the activity were documented; wbPA was defined as activities involving standing, walking, running, and extraneous weights. The average hours per day doing wbPA at work, during commute, and for leisure were calculated from the weekly frequency and duration per day.

#### Anthropometric data

Weight was measured to the nearest 0.1 kg with a digital SECA balance, and standing height was measured to the nearest 1 mm with a plastic stadiometer (Leicester height measure; Chasmors Ltd). Measurements were taken twice, and the average of 2 values was used in the analysis. BMI was calculated as weight (kg)/height (m)^2^.

#### Serum 25(OH)D

Assays were conducted on fasting venous samples at the National Institute of Nutrition. Serum 25-hydroxyvitamin D_2_ and 25-hydroxyvitamin D_3_ were extracted in quantitative HPLC assays and detected at 265 nm by using an ultraviolet detector (CV: 7%).

#### Dual-energy X-ray absorptiometry

Bone mass, lean mass, and fat mass measurements were assessed with dual-energy X-ray absorptiometry (DXA): 91% of scans were performed with a Hologic Discovery A model and 9% with a Hologic 4500W. The same software version was used on both machines. On the basis of repeated measurements for 30 participants with Hologic Discovery A, the CVs were determined to be 0.7% for hip bone mineral density (BMD), 1.3% for LS BMD, and 0.9% for whole-body BMD. The whole-body scan was performed while the participant lay supine on the scanning bed with their arms resting by their sides. Women suspected of pregnancy (*n* = 26) were excluded from DXA scanning. Standard Hologic software options were used to define regions of the body (head, arms, trunk, and legs). Scans were coded for artifacts by a visual inspection, and those with major movement and incomplete scans were excluded. For LS scans, pathological changes such as osteoarthritis affecting ≥2 vertebrae were excluded; if only one vertebra was affected, the scan was reanalyzed after the affected part was excluded (28). BMD (g/cm^2^) was calculated for total hip, LS (L1–L4). Whole-body BMD was included for analyses in men only, because most women wore bangles and other jewelry, which were counted as major artifacts. Whole-body lean mass (kg) and fat mass (kg) were used for the analyses.

### Quality control

We produced detailed protocols and regularly checked compliance to standardize the work of the fieldwork team. The anthropometric equipment was calibrated at the start of every clinic. The BMD estimation process was automated in software, which reduces the potential for bias arising from the DXA technician, who knew the intervention group assignment. Hip and LS DXA scans were analyzed by a single trained technician. For quality assurance of DXA scans, a spine phantom was scanned every day to check for acceptable ranges.

### Statistical analysis

Descriptive statistics were calculated for each sex. The associations of hip and LS BMD with early-life supplementation and current risk factors were modeled in multilevel regression models that accounted for sibling pairs and village clusters (284 sibling pairs from 29 villages). All multilevel models also adjusted for DXA scanner types. Variables with positively skewed distributions were logarithmically transformed. The analysis was done on an intent-to-treat basis by using the area of birth as proxy for intervention and control villages. List-wise deletion was used to handle the missing data.

Current risk factors included in the multilevel regression models were age, sex, height, fat mass, lean mass, household SLI, serum 25(OH)D, wbPA, and dietary intake of fruit and vegetables, calcium, protein, and total calories. Three models were fitted for each of the 2 outcome variables (hip and LS BMD) to incrementally adjust for various risk factors: model 1 (age and sex), model 2 (age, sex, anthropometric, and socioeconomic factors, eg, height, fat mass, lean mass, and SLI), and model 3: age, sex, anthropometric and socioeconomic factors, serum 25(OH)D, dietary factors (fruit and vegetable, calcium, protein, and energy intakes), and wbPA.

Interaction terms for sex and supplementation and for sex and wbPA were examined, and a robust interaction term between sex and supplementation was found for hip BMD (*P* = 0.03 for hip, 0.61 for LS). Sex-stratified multilevel regression analysis was performed for all models. The intraclass correlation analysis for model 3 showed that between-village variability in the outcome was quite small compared with between-sibling variability (data not shown). All analyses were conducted by using R, version 2.15.2, and multilevel modeling was done with nlme version 3.1–105.

## RESULTS

Of the original Hyderabad Nutrition Trial cohort, all 2601 participants born between 1987 and 1990 were invited to the clinic, 1446 (56%) of whom accepted the invitation and attended. The participants in the study were more likely to be men than were the eligible nonparticipants (*see* Supplemental Table 1 under “Supplemental data” in the online issue), partially attributable to the emigration of women because of marriage. DXA scans without major artifacts were available in 1351 (93%) participants for hip and 1360 (94%) for LS. Serum 25(OH)D data were available for 1037 (71%) participants. Information on the other variables including SLI, dietary intake estimates, and physical activity were available for ≥98% of the participants.

The key characteristics of the participants are shown in **Table 1**. These young adults were generally lean (BMI: 19.5). The median total energy intake was 2735 kcal/d. Although Indian standards were unavailable and caution should be exercised in applying standards across populations, 57% of the participants had low serum 25(OH)D concentrations (<20 ng/mL) (29) and 70% of the participants had a low fruit and vegetable intake (<400 g/d) (30) according to international standards. Women were more likely to be unemployed than were men, with most engaging in household work (*n* = 166), whereas men were more likely to be students or skilled manual laborers. Women spent fewer hours on wbPA at work, during commuting, or for leisure. Tobacco and alcohol use were rare among women, whereas approximately one-fifth of male participants reported current tobacco or alcohol use.

**TABLE 1:** Participant characteristics of the Andhra Pradesh Children and Parents Study cohort in 2009–2010*^1^*

	Women	Men
Total subjects [*n* (%)]	465 (32.2)	981 (67.8)
Age (y)	20.44 ± 1.2*^2^*	20.2 ± 1.2
Height (cm)	152.64 ± 5.3	166.67 ± 6.2
Weight (kg)	44.61 ± 7.5	54.81 ± 8.7
BMI (kg/m^2^)	19.12 ± 2.9	19.71 ± 2.8
Lean mass (kg)	29.93 ± 3.9	43.42 ± 5.5
Fat mass (kg)	12.2 (11.8, 12.6)*^3^*	9.1 (8.9, 9.3)
SLI	17.62 ± 4.5	18.65 ± 4.2
Education [*n* (%)]		
None/primary only	131 (28.2)	171 (17.5)
Secondary	305 (65.6)	751 (76.6)
Beyond secondary	29 (6.2)	58 (5.9)
Occupation [*n* (%)]		
Unemployed	170 (36.6)	28 (2.8)
Student	143 (30.8)	399 (40.3)
Manual work	90 (19.4)	188 (19)
Skilled manual work	48 (10.3)	303 (30.6)
Professional	14 (3)	72 (7.3)
Serum 25(OH)D (ng/mL)	18.52 (17.8, 19.3)	23.12 (22.3, 24)
Dietary intake		
Calcium (mg/d)	423.8 (405, 443.5)	618.7 (600.3, 637.6)
Protein (g/d)	47.5 (46.1, 49)	75.2 (73.5, 76.9)
Energy (kcal/d)	1987.4 (1930.9, 2045.6)	3107.9 (3042.8, 3174.5)
Fruit and vegetables (g/d)	210 (200, 220.5)	341.1 (329, 353.8)
Dairy products (g/d)	118.8 (106.6, 132.4)	159.1 (149.4, 169.5)
Lifestyle		
Tobacco use [*n* (%)]	0 (0)	188 (19.2)
Alcohol use [*n* (%)]	8 (1.7)	217 (22.1)
wbPA (h)	0.71 (0.63, 0.79)	1.9 (1.8, 2)

1 SLI, Standard of Living Index; wbPA, weight-bearing physical activity; 25(OH)D, 25-hydroxyvitamin D.

2 Mean ± SD (all such values).

3 Geometric mean; geometric 95% CI in parentheses (all such values; for variables with a skewed distribution).

Hip and LS BMD values in this population were generally low when compared with the reference values for the Indian population (31) (**Table 2**). In multivariable models adjusted for current risk factors, early-life supplementation was not positively associated with BMD (Table 2). For men, the negative association between early-life supplementation and BMD remained robust in the fully adjusted model for hip BMD, whereas this association was attenuated when the serum 25(OH)D concentration was added to the model for LS BMD. Of the current risk factors assessed in model 3, height and lean mass were positively associated with hip, LS, and whole-body BMDs (**Table 3, Table 4, and Table 5**). Fat mass was positively associated with LS BMD in women and whole-body BMD in men. The positive associations between hip BMD and wbPA remained robust when fat mass was added to the models but became attenuated with the addition of lean mass (*see* Supplemental Table 2 under “Supplemental data” in the online issue). The serum 25(OH)D concentration was not strongly associated with BMD in the multivariable models. The positive associations of dietary intake of calcium, protein, and total calories with BMD were attenuated in the fully adjusted models, except for calcium and hip BMD in women.

**TABLE 2:** Multilevel models examining the association between supplemental nutrition and BMD in the Andhra Pradesh Children and Parents Study cohort in 2009–2010*^1^*

	BMD*^2^*		Early-life supplementation effect*^3^*^,^*^4^*		
	Intervention	Control	Model 1	Model 2	Model 3
	*g/cm^2^*	*g/cm^2^*			
Hip					
Women	0.83 ± 0.09	0.84 ± 0.1	−0.005 (−0.025, 0.015)	0.003 (−0.014, 0.021)	0.004 (−0.015, 0.022)
* P*			0.62	0.7	0.7
Men	0.93 ± 0.11	0.96 ± 0.12	−0.03 (−0.046, −0.015)	−0.02 (−0.033, 0.006)	−0.02 (−0.041, −0.007)
* P*			<0.001	0.006	0.009
LS					
Women	0.86 ± 0.1	0.86 ± 0.1	−0.003 (−0.025, 0.018)	−0.002 (−0.021, 0.017)	−0.005 (−0.025, 0.016)
*P*			0.76	0.85	0.65
Men	0.92 ± 0.1	0.94 ± 0.11	−0.02 (−0.037, −0.003)	−0.01 (−0.032, 0.004)	−0.005 (−0.03, 0.02)
* P*			0.02	0.12	0.68
WB					
Men	1.06 ± 0.08	1.07 ± 0.08	−0.02 (−0.029, −0.002)	−0.007 (−0.021, 0.006)	−0.012 (−0.034, 0.009)
* P*			0.03	0.27	0.25

1 Sample sizes: women (hip BMD intervention, *n* = 413; control, *n* = 196; LS BMD intervention, *n* = 412; control, *n* = 412) and men (hip BMD intervention, *n* = 480; control, *n* = 466; LS BMD intervention, *n* = 486; control, *n* = 471; WB BMD intervention, *n* = 458; control, *n* = 444). BMD, bone mineral density; LS, lumbar spine; SLI, Standard of Living Index (tertiles: low, 0–16; middle, 17–20; high, 21–32); WB, whole body.

2 Values are means ± SDs.

3 Values are β coefficients; 95% CIs in parentheses.

4 Model 1: hip BMD (*n* = 368 women, *n* = 838 men); LS (*n* = 366 women, *n* = 850 men); and WB (*n* = 808 men). Model 2: hip BMD (*n* = 366 women, *n* = 833 men); LS (*n* = 364 women, *n* = 845 men); and WB (*n* = 804 men). Model 3: hip BMD (*n* = 329 women, *n* = 535 men); LS (*n* = 326 women, *n* = 538 men); and WB (*n* = 515 men). Models 1–3 are multilevel regression models accounting for villages and sibling effects and adjusted for types of dual-energy X-ray absorptiometry machines in addition to the following variables: model 1 [adjusted for age (y)], model 2 [adjusted for age, height (cm), lean mass (kg), fat mass (kg), and SLI (tertiles)], and model 3 [adjusted for age, height, lean mass, fat mass, SLI, serum 25-hydroxyvitamin D (ng/mL), calcium intake (mg/d), protein intake (g/d), energy intake (kcal/d), and weight-bearing physical activity (h/d)].

**TABLE 3:** Women: univariable and multivariable models examining current risk factors of hip and lumbar spine BMD in the Andhra Pradesh Children and Parents Study cohort in 2009–2010*^1^*

	Hip BMD (*n* = 329)				Lumbar spine BMD (*n* = 326)			
Univariable		Multivariable*^2^*		Univariable		Multivariable*^2^*	
	β coefficient (95% CI)	*P*	β coefficient (95% CI)	*P*	β coefficient (95% CI)	*P*	β coefficient (95% CI)	*P*
Age (y)	0.005 (−0.003, 0.012)	0.25	0.001 (−0.007, 0.008)	0.89	−0.001 (−0.008, 0.007)	0.89	−0.005 (−0.013, 0.004)	0.28
SLI low*^3^*	−0.029 (−0.054, −0.005)	0.022	−0.017 (−0.043, 0.009)	0.19	−0.044 (−0.07, −0.019)	0.001	−0.034 (−0.062, −0.007)	0.019
SLI mid*^3^*	−0.029 (−0.054, −0.005)	0.007	−0.019 (−0.044, 0.006)	0.13	−0.044 (−0.07, −0.019)	0.004	−0.031 (−0.057, −0.004)	0.028
Height (cm)	0.003 (0.001, 0.005)	0.001	−0.002 (−0.004, 0)	0.094	0.005 (0.003, 0.006)	<0.001	0.002 (0, 0.004)	0.077
Fat mass (kg)	0.082 (0.053, 0.111)	<0.001	0.01 (−0.027, 0.047)	0.6	0.088 (0.058, 0.118)	<0.001	0.053 (0.014, 0.093)	0.012
Lean mass (kg)	0.011 (0.009, 0.013)	<0.001	0.011 (0.008, 0.014)	<0.001	0.008 (0.006, 0.011)	<0.001	0.004 (0, 0.008)	0.04
Vitamin D (ng/mL)	0.023 (−0.002, 0.048)	0.067	0.02 (−0.002, 0.043)	0.085	0.004 (−0.022, 0.03)	0.74	0.003 (−0.022, 0.027)	0.84
Fruit and vegetables (g)	0.009 (−0.007, 0.025)	0.25	−0.004 (−0.026, 0.018)	0.72	0.014 (−0.002, 0.031)	0.089	0.001 (−0.022, 0.025)	0.9
Calcium (mg/d)	0.017 (0, 0.033)	0.052	0.036 (0.003, 0.069)	0.037	0.016 (–0.001, 0.033)	0.074	0.01 (−0.026, 0.046)	0.57
Protein (g/d)	0.012 (−0.014, 0.037)	0.37	−0.087 (−0.22, 0.044)	0.19	0.021 (−0.005, 0.047)	0.11	−0.04 (−0.181, 0.1)	0.57
Energy (kcal)	0.009 (−0.01, 0.029)	0.35	0.042 (−0.079, 0.16)	0.486	0.016 (−0.004, 0.036)	0.12	0.031 (−0.097, 0.16)	0.63
wbPA (h)	0.02 (0.001, 0.039)	0.042	0.014 (−0.006, 0.033)	0.161	0.012 (−0.008, 0.031)	0.23	0.016 (−0.005, 0.037)	0.13

1 All models were adjusted for type of dual-energy X-ray absorptiometry machine in multilevel regression models accounting for village clusters and sibling pairs. Multivariable models were additionally adjusted for age, SLI, height, fat mass, lean mass, serum 25-hydroxyvitamin D, wbPA, and dietary intake of fruit and vegetables, calcium, protein, and energy. Serum 25-hydroxyvitamin D, fruit and vegetable intake, calcium intake, protein intake, energy intake, and wbPA were log transformed to account for skewed distributions. BMD, bone mineral density (g/cm^2^); mid, middle; SLI, Standard of Living Index; wbPA, weight-bearing physical activity.

2 Based on model 3.

3 SLI tertiles: low, 0–16; middle, 17–20; high, 21–32.

**TABLE 4:** Men: univariable and multivariable models examining current risk factors of hip and lumbar spine BMD in the Andhra Pradesh Children and Parents Study cohort in 2009–2010*^1^*

	Hip BMD (*n* = 535)				Lumbar spine BMD (*n* = 538)			
Univariable		Multivariable*^2^*		Univariable		Multivariable*^2^*	
	β coefficient (95% CI)	*P*	β coefficient (95% CI)	*P*	β coefficient (95% CI)	*P*	β coefficient (95% CI)	*P*
Age (y)	0.006 (0, 0.013)	0.049	−0.002 (−0.008, 0.005)	0.66	0.006 (0, 0.012)	0.036	0.002 (−0.005, 0.009)	0.62
SLI low*^3^*	−0.01 (−0.028, 0.008)	0.29	−0.00005 (−0.021, 0.021)	0.996	0.005 (−0.012, 0.022)	0.54	0.013 (−0.008, 0.034)	0.23
SLI mid*^3^*	−0.01 (−0.028, 0.008)	0.067	−0.003 (−0.023, 0.017)	0.76	0.005 (−0.012, 0.022)	0.99	0.013 (−0.007, 0.033)	0.2
Height (cm)	0.003 (0.002, 0.004)	<0.001	−0.004 (−0.005, −0.002)	<0.001	0.002 (0.001, 0.003)	<0.001	−0.002 (−0.003, 0)	0.037
Fat mass (kg)	0.074 (0.056, 0.091)	<0.001	−0.027 (−0.053, −0.002)	0.041	0.052 (0.035, 0.068)	<0.001	−0.01 (−0.036, 0.016)	0.47
Lean mass (kg)	0.01 (0.009, 0.011)	<0.001	0.012 (0.01, 0.015)	<0.001	0.007 (0.006, 0.008)	<0.001	0.008 (0.005, 0.01)	<0.001
Vitamin D (ng/mL)	0.024 (0.003, 0.046)	0.029	0.016 (−0.003, 0.035)	0.096	0.023 (0.002, 0.043)	0.029	0.015 (−0.005, 0.034)	0.14
Fruit and vegetables (g)	0.027 (0.015, 0.039)	<0.001	0.001 (−0.018, 0.02)	0.94	0.018 (0.008, 0.029)	0.001	0.012 (−0.006, 0.031)	0.2
Calcium (mg/d)	0.025 (0.012, 0.039)	<0.001	−0.021 (−0.053, 0.01)	0.18	0.015 (0.003, 0.027)	0.018	−0.011 (−0.042, 0.02)	0.5
Protein (g/d)	0.058 (0.039, 0.076)	<0.001	0.037 (−0.068, 0.143)	0.49	0.036 (0.019, 0.052)	<0.001	0.029 (−0.081, 0.138)	0.6
Energy (kcal)	0.042 (0.025, 0.058)	<0.001	0.005 (−0.093, 0.103)	0.92	0.022 (0.009, 0.036)	0.001	−0.02 (−0.121, 0.082)	0.7
wbPA (h)	0.04 (0.025, 0.054)	<0.001	0.017 (0, 0.033)	0.051	0.031 (0.017, 0.045)	<0.001	0.02 (0.004, 0.037)	0.02

1 All models were adjusted for type of dual-energy X-ray absorptiometry machine in multilevel regression models accounting for village clusters and sibling pairs. Multivariable models were additionally adjusted for age, SLI, height, fat mass, lean mass, serum 25-hydroxyvitamin D, wbPA, and dietary intake of fruit and vegetables, calcium, protein, and energy. Serum 25-hydroxyvitamin D, fruit and vegetable intake, calcium intake, protein intake, energy intake, and wbPA were log transformed to account for skewed distributions. BMD, bone mineral density (g/cm^2^); mid, middle; SLI, Standard of Living Index; wbPA, weight-bearing physical activity.

2 Based on model 3.

3 SLI tertiles: low, 0–16; middle, 17–20; high, 21–32.

**TABLE 5:** Men: univariable and multivariable models examining current risk factors of whole-body BMD in the Andhra Pradesh Children and Parents Study cohort in 2009–2010*^1^*

	Whole-body BMD (*n* = 562)			
Univariable		Multivariable*^2^*	
	β coefficient (95% CI)	*P*	β coefficient (95% CI)	*P*
Age (y)	0.011 (0.007, 0.016)	<0.001	0.007 (0.002, 0.012)	0.012
SLI low*^3^*	0.0003 (−0.013, 0.013)	0.97	0.011 (−0.005, 0.026)	0.17
SLI mid*^3^*	−0.0008 (−0.013, 0.013)	0.9	0.009 (−0.005, 0.023)	0.22
Height (cm)	0.002 (0.001, 0.003)	<0.001	−0.002 (−0.003, −0.001)	0.001
Fat mass (kg)	0.026 (0.014, 0.039)	<0.001	−0.043 (−0.062, −0.024)	<0.001
Lean mass (kg)	0.006 (0.005, 0.007)	<0.001	0.008 (0.007, 0.01)	<0.001
Vitamin D (ng/mL)	0.024 (0.008, 0.039)	0.004	0.014 (0, 0.029)	0.057
Fruit and vegetables (g)	0.017 (0.008, 0.025)	<0.001	0.002 (−0.012, 0.016)	0.76
Calcium (mg/d)	0.02 (0.01, 0.03)	<0.001	−0.0003 (0.023, 0.023)	0.98
Protein (g/d)	0.035 (0.022, 0.049)	<0.001	0.047 (−0.032, 0.127)	0.24
Energy (kcal)	0.024 (0.013, 0.036)	<0.001	−0.039 (−0.112, 0.035)	0.3
wbPA (h)	0.022 (0.012, 0.033)	<0.001	0.013 (0.001, 0.025)	0.043

1 All models were adjusted for type of dual-energy X-ray absorptiometry machine in multilevel regression models accounting for village clusters and sibling pairs. Multivariable models were additionally adjusted for age, SLI, height, fat mass, lean mass, serum 25-hydroxyvitamin D, wbPA, and dietary intake of fruit and vegetables, calcium, protein, and energy. Serum 25-hydroxyvitamin D, fruit and vegetable intake, calcium intake, protein intake, energy intake, and wbPA were log transformed to account for skewed distributions. BMD, bone mineral density (g/cm^2^); mid, middle; SLI, Standard of Living Index; wbPA, weight-bearing physical activity.

2 Based on model 3.

3 SLI tertiles: low, 0–16; middle, 17–20; high, 21–32.

## DISCUSSION

Young adults from this transitional rural community were generally lean, and their BMD was low. No strong evidence of an association was found between BMD and early-life supplementation. Current height, BMI, and wbPA were associated with bone mass, whereas no clear evidence of an association with bone mass was found for other current risk factors, including social position and dietary intakes of calcium, protein, and calories. Strong evidence of an association was found between BMD and lean mass.

### Comparison with previous research

The Guatemala trial randomized 4 villages within pairs to offer either low-energy (1.38 MJ) or high-energy (3.76 MJ, proteins, and micronutrients) supplements to pregnant women and children until the age of 7 y (7). Follow-up in adolescence (mean age: 16.7 y) showed a positive association between bone mass and protein-calorie supplementation, but this association was not statistically robust when the models were adjusted for current weight and stature. In an urban cohort of participants from India (the New Delhi Birth Cohort study), bone mass at age 33–39 y was positively associated with birth weight (proxy measure for early-life undernutrition), but this association was also attenuated to null on adjustment for current anthropometric and lifestyle risk factors (32). In our study, no strong evidence of a positive association was found between bone mass and early-life nutritional supplementation; if anything, there was weak evidence of an inverse association between the two. Higher osteoporotic fracture rates are observed in urban areas in high-income countries, which may be partially because of the more sedentary lifestyles among city dwellers (3). Anecdotal evidence suggests that urbanization of the study area over the past decade may have proceeded more rapidly in the intervention villages than in the control villages. In the first follow-up study in 2003–2005, we observed taller statures in children from the intervention villages, raising the possibility of a positive effect of early-life supplementation on skeletal growth during early adolescence. Any positive effects of early-life supplementation during early adolescence may have been negated as a result of changes in lifestyles during late adolescence and young adulthood in more urbanized villages. Higher rates of physical activity may be associated with more rural lifestyles (eg, agricultural occupations) in the control villages, which may partially explain the inverse association between early-life supplementation and BMD; however, our study did not have appropriate measures to examine this possibility definitively. Inclusion of population size as a marker of urbanization did not materially change our results.

Current wbPA and height and lean mass were strong determinants of bone mass in this study, consistent with the findings of previous studies that examined bone mass in individuals in the growth phase (11, 32–35). Fat and lean mass, 2 main components of body mass, have been suggested, although not consistently, as independent determinants of BMD (13, 36, 37). Our analyses of relative contributions of fat and lean mass to BMD showed clear evidence of a positive association between BMD and lean mass, but not fat mass. A similar analysis from Thailand suggested that uniformly low body fat, a common characteristic observed in rural areas of low- and middle-income countries, may obscure the association between BMD and fat mass because of a lack of sufficient variability (ie, low study power) (13). The addition of lean mass to the models attenuated the association between BMD and wbPA, which suggests that this association may be partially mediated through lean mass accumulation. On the other hand, lean mass as a surrogate for physical activity may be measured with relatively less error as compared with physical activity assessed by questionnaire, which further supports the role of physical activity in bone mass accrual. Our finding on physical activity is particularly important for this transitional rural community because physical inactivity has been suggested to partially explain the association between lower bone mass and urbanization (1, 4, 38, 39).

The magnitudes of the effects of dietary calcium intake have varied in previous studies (40–42), and it is possible that calcium intake may only be positively associated with bone mass in individuals with very low vitamin D concentrations (12). In this study population, no evidence of an association was found between BMD and dietary calcium, even though the serum 25(OH)D concentration was fairly low (43, 44). Studies have shown a positive association between bone mass and protein intake, although a high protein intake has been suggested to be both detrimental and protective for bones (17, 45). The extremely low energy intake of adolescents may be associated with low BMD, but the effect of a high calorie intake in healthy young adults is unclear (46, 47). Our results showed no evidence of an association between BMD and protein or energy intake. Fruit and vegetable (Tables 3, 4, and 5) and dairy product (data not shown) intakes have also been suggested as potential determinants of bone mass, but no associations were observed in this study (4, 48).

### Strengths and limitations

The main strength of this study was the combined assessment of effects of both early-life and current risk factors on BMD. Compared with observational studies using birth weight as a proxy measure for early-life nutrition, the initial supplementation trial with a controlled design in our study should have reduced the chances of confounding by other factors and more directly assessed the effects of early-life nutrition on bone mass accrual. The rapid but uneven socioeconomic development of the area over the past decade has also introduced remarkable changes in the current lifestyles of the study population, providing sufficient variation in these exposures for their effects to be robustly examined. The study population size was large compared with other DXA bone studies in young adults from low- and middle-income countries, which added further strength to the study.

The study also had some limitations. The DXA facility location was not convenient or feasible to reach for some participants, and the response rate of participants invited to clinic was 56% of the original study sample. The response rates were similar between intervention and control villages, but the possibility of selection bias could not be ruled out. Women were overrepresented among the nonparticipants as a result of migration due to marriage. We did not have direct data on nutritional supplementation; mothers in the intervention area were presumed to have taken the supplement, which could also have been shared with other family members. As a result, the dose of nutritional supplement may have been too modest for persistent effects on bone development. On the other hand, our results mimicked real-life circumstances, which provided plausible estimates of the long-term effects of nutritional supplementation programs. Although we collected detailed information on types and amounts of physical activity, we did not have information on the amounts of loads that each type of physical activity applied to bones. Finally, the cross-sectional assessment of bone mass did not allow us to examine the causality between these exposures and outcomes.

### Implications

Bone remodeling is influenced by many factors throughout life. In emerging-economy countries, many rural communities are experiencing the effects of rapid economic development superimposed on early disadvantages. Whereas improved nutrition may be able to mitigate some of the effects of early-life undernutrition, more urbanized lifestyles may also have negative effects on bone mass accrual in the future. Physical activity may be incorporated into daily lives relatively cheaply and easily and is also important for the prevention of other NCDs, such as cardiovascular diseases (49). Our findings provide yet another reason for promoting physical activity in rural communities in low- and middle-income countries that are undergoing rapid urbanization.

## Supplementary Material

Supplemental data

## Footnotes

4 Abbreviations used: BMD, bone mineral density; DXA, dual-energy X-ray absorptiometry; LS, lumbar spine; NCD, noncommunicable disease; SLI, Standard of Living Index; wbPA, weight-bearing physical activity; 25(OH)D, 25-hydroxyvitamin D.
