# Supplementary material for: Life-course determinants of bone mass in young adults from a transitional rural community in India: the Andhra Pradesh Children and Parents Study (APCAPS)1
Source: Am J Clin Nutr. 2014 Apr 2;99(6):1450–9. doi: 10.3945/ajcn.113.068791 (PMC4021785; doi:10.3945/ajcn.113.068791)
Supplement: Supplemental data [file supp_99_6_1450__index.html]

Supplemental data 

# Life-course determinants of bone mass in young adults from a transitional rural community in India: the Andhra Pradesh Children and Parents Study (APCAPS)

## Supplemental data

**Files in this Data Supplement:**

- Supplemental data - Tables 1 and 2
