# Supplementary material for: Life-course determinants of bone mass in young adults from a transitional rural community in India: the Andhra Pradesh Children and Parents Study (APCAPS)1
Source: Am J Clin Nutr. 2014 Apr 2;99(6):1450–9. doi: 10.3945/ajcn.113.068791 (PMC4021785; doi:10.3945/ajcn.113.068791)
Supplement: Supplemental data [file 113.068791_ajcn068791SupplementaryData1.doc]

**Online Supplemental Tables**

**Table 1** Characteristics of young adults who attended and those who did not attend clinics at the 2009-2010 study of the Andhra Pradesh Children and Parents Study. Values are numbers (percentages) unless stated otherwise.

| **Characteristic** | **Intervention area (n = 1342)** | | | **Control area (n = 1259)** | | | |
| --- | --- | --- | --- | --- | --- | --- | --- |
|  | **Participants**  **(n=738)** | **Non-participants**  **(n=604)** | **p*** | **Participants**  **(n = 708)** | **Non-participants**  **(n = 551)** | | **p*** |
| Mean(SD) age (years)^†^ | 20.7(1.1) | 20.7 (1.1) | 0.12 | 20.7 (1.1) | 20.7 (1.1) | | 0.43 |
| Women | 236 (32) | 423 (70) | <0.001 | 223 (31.5) | 399 (72.4) | | <0.001 |
| Occupation^‡^ | (n = 737) | (n = 598) | <0.001 | (n = 695) | (n = 532) | | <0.001 |
| Full time student | 608 (82.5) | 415 (69.4) |  | 534 (76.8) | 328 (60.3) | |  |
| Full time employment | 90 (12.2) | 119 (19.9) |  | 124 (17.8) | 155 (28.5) | |  |
| Other (neither, both) | 39 (5.3) | 64 (10.7) |  | 37 (5.3) | 1. (9) | |  |
| Birth weight (mg) | (n=198) | (n=136) | 0.74 | (n=273) | (n=165) | 0.26 | |
|  | 2715.6(416.5) | 2730.3(381.4) |  | 2639.6(432.4) | 2592.4(426.7) |  | |

* These p-values are based on unpaired t tests or Χ^2^ tests for heterogeneity with appropriate degrees of freedom.

† As of January 1 2009.

‡ Based on 2003 data.

**Table 2:** Models with or without lean mass examining associations of weight-bearing physical activity with hip and lumbar spine BMD of the Andhra Pradesh Children and Parents Study cohort in 2009-2010. Values are β coefficient (95% CI) and p-values.

|  | Hip BMD | | | | Lumbar spine BMD | | | |
| --- | --- | --- | --- | --- | --- | --- | --- | --- |
|  | Model 3 without lean mass | | Model 3 | | Model 3 without lean mass | | Model 3 | |
|  | β coefficient | p | β coefficient | p | β coefficient | p | β coefficient | p |
| **Women** |  |  |  |  |  |  |  |  |
| Fat mass (kg) | 0.078  (0.047 to 0.11) | <0.001 | 0.009  (-0.027 , 0.047) | 0.59 | 0.077  (0.045, 0.11) | <0.001 | 0.053  (0.014 , 0.093) | 0.01 |
| Lean mass (kg) |  |  | 0.011  (0.008 , 0.014) | <0.001 |  |  | 0.004  (0 , 0.008) | 0.04 |
| wbPA (hour) | 0.024  (0.004, 0.045) | 0.021 | 0.014  (-0.006 , 0.033) | 0.16 | 0.02  (-0.001, 0.04) | 0.06 | 0.016  (-0.004 , 0.037) | 0.12 |
| **Men** |  |  |  |  |  |  |  |  |
| Fat mass (kg) | 0.053  (0.047, 0.11) | <0.001 | -0.028  (-0.053 , -0.002) | 0.04 | 0.042  (0.02, 0.063) | <0.001 | -0.009  (-0.035 , 0.017) | 0.49 |
| Lean mass  (kg) |  |  | 0.012  (0.01 , 0.015) | <0.001 |  |  | 0.008  (0.005 , 0.01) | <0.001 |
| wbPA (hour) | 0.028  (0.004, 0.045) | 0.003 | 0.016  (0 , 0.033) | 0.06 | 0.028  (0.011, 0.045) | 0.002 | 0.02  (0.003 , 0.036) | 0.02 |

Sample size: Hip BMD Women n=329, Men n=535; Lumbar spine BMD Women n=326, Men n=538.

BMD: Bone mineral density (g/cm^2^); LS: Lumbar spine; wbPA: Weight bearing physical activity

All models adjusted for DXA machine types in multilevel models accounting for village clusters and sibling pairs. Multivariable models additionally adjusted for early life supplementation, age, height, SLI, dietary intake (fruit and vegetable, calcium, protein, calories), and serum vitamin D.
